# Human Retinal Transmitochondrial Cybrids with J or H mtDNA Haplogroups Respond Differently to Ultraviolet Radiation: Implications for Retinal Diseases

**DOI:** 10.1371/journal.pone.0099003

**Published:** 2014-06-11

**Authors:** Deepika Malik, Tiffany Hsu, Payam Falatoonzadeh, Javier Cáceres-del-Carpio, Mohamed Tarek, Marilyn Chwa, Shari R. Atilano, Claudio Ramirez, Anthony B. Nesburn, David S. Boyer, Baruch D. Kuppermann, S. Michal Jazwinski, Michael V. Miceli, Douglas C. Wallace, Nitin Udar, M. Cristina Kenney

**Affiliations:** 1 Gavin Herbert Eye Institute, University California Irvine, Irvine, California, United States of America; 2 Department of Ophthalmology, El-Minya University, El-Minya, Egypt; 3 Cedars-Sinai Medical Center, Los Angeles, California, United States of America; 4 Retina-Vitreous Associates Medical Group; Beverly Hills, California, United States of America; 5 Tulane Center for Aging, Tulane University, New Orleans, Louisiana, United States of America; 6 Center for Mitochondrial and Epigenomic Medicine, Children’s Hospital of Philadelphia and Department of Pathology and Laboratory Medicine, University of Pennsylvania, Philadelphia, Pennsylvania, United States of America; 7 Department of Pathology and Laboratory Medicine, University California Irvine, Irvine, California, United States of America; Schepens Eye Research Institute/Massachusetts Eye and Ear, Department of Ophthalmology, Harvard Medical School, Boston, MA, United States of America

## Abstract

**Background:**

It has been recognized that cells do not respond equally to ultraviolet (UV) radiation but it is not clear whether this is due to genetic, biochemical or structural differences of the cells. We have a novel cybrid (cytoplasmic hybrids) model that allows us to analyze the contribution of mitochondrial DNA (mtDNA) to cellular response after exposure to sub-lethal dose of UV. mtDNA can be classified into haplogroups as defined by accumulations of specific single nucleotide polymorphisms (SNPs). Recent studies have shown that J haplogroup is high risk for age-related macular degeneration while the H haplogroup is protective. This study investigates gene expression responses in J cybrids versus H cybrids after exposure to sub-lethal doses of UV-radiation.

**Methodology/Principal Findings:**

Cybrids were created by fusing platelets isolated from subjects with either H (n = 3) or J (n = 3) haplogroups with mitochondria-free (Rho^0^) ARPE-19 cells. The H and J cybrids were cultured for 24 hours, treated with 10 mJ of UV-radiation and cultured for an additional 120 hours. Untreated and treated cybrids were analyzed for growth rates and gene expression profiles. The UV-treated and untreated J cybrids had higher growth rates compared to H cybrids. Before treatment, J cybrids showed lower expression levels for CFH, CD55, IL-33, TGF-A, EFEMP-1, RARA, BCL2L13 and BBC3. At 120 hours after UV-treatment, the J cybrids had decreased CFH, RARA and BBC3 levels but increased CD55, IL-33 and EFEMP-1 compared to UV-treated H cybrids.

**Conclusion/Significance:**

In cells with identical nuclei, the cellular response to sub-lethal UV-radiation is mediated in part by the mtDNA haplogroup. This supports the hypothesis that differences in growth rates and expression levels of complement, inflammation and apoptosis genes may result from population-specific, hereditary SNP variations in mtDNA. Therefore, when analyzing UV-induced damage in tissues, the mtDNA haplogroup background may be important to consider.

## Introduction

Age-related macular degeneration (AMD) is a multifactorial disease, wherein multiple genetic variants and environmental risk factors, including diet, smoking, hypertension, light exposure, ultraviolet (UV) radiation, blue light and ionizing radiation, contribute to the disease [1]. Despite new medical and surgical interventions, AMD is one of the important causes of decreased vision in the United States. According to the National Health And Nutrition Examination Survey (NHANES 2005–2008 report), the estimated prevalence of any AMD was 6.5% and of late AMD was 0.8% in the US population 40 years and older [2]. Aging and oxidative damage to retinal pigment epithelial (RPE) cells, a monolayer of cells located between the neural retina and Bruch’s membrane contributes to AMD [3]. Early AMD has sub-retinal drusen, and can progress to advanced AMD, which includes dry (non-exudative, atrophic) and wet (exudative, neovascular) forms [4]. The dry form of advanced AMD is characterized by geographic atrophy involving the macula and accounts for approximately 25% of cases with central vision loss [5]. The wet form accounts for 75% of the central loss of vision and is characterized by choroidal neovascularization and if untreated, eventually a disciform scar in the macular region [5].

Solar radiation consists of variable wavelengths: UV-C (100–280 nm), UV-B (280–315 nm), UV-A (315–400 nm) and visible spectrum (400–700 nm) [6]. Light with shorter wavelengths has higher energy and can produce intense tissue damage. The human cornea filters out most of the radiation below 295 nm [7]. The human lens in younger individuals can transmit UV-B radiation but by adulthood, the lens blocks almost all UV-A and UV-B and most of the blue light [8]. However, exposure to both UV-A and UV-B are known to produce cataract and UV-radiation imposes increased damage to both pediatric and adult retina. Damage induced by UV-radiation is cumulative and depends on the duration, wavelength and intensity of exposure.

After 3 years of age, the yellow pigment 3-hydroxykynurenine, a tryptophan metabolite, gradually develops in the human lens and absorbs UV-radiation, which provides protection to the retina. Lutein and zeaxanthin are macular pigments and along with glutathione protect the retina against inflammatory and photo-oxidative damage. Antioxidants, antioxidant enzymes, and melanin pigments within RPE cells and the choroid provide protection against light-induced ocular damage. However, with age, these pigments and antioxidants are gradually depleted, thereby making the retina more susceptible to free radical damage. Acute exposure to UV-radiation, as in sunlight reflected from snow or an eclipse, can produce inflammatory damage and interleukin responses [9], [10]. Over a lifetime, chronic exposure induces accumulative photo-oxidative damage via singlet oxygen and free radical production that leads to damage of DNA, proteins and lipids.

Aging is associated with increased DNA damage, including both mitochondrial (mtDNA) and nuclear DNA (nDNA), and decreased efficacy of the DNA repair mechanisms [11]. The DNA repair process becomes less efficient in presence of risk factors such as UV exposure and environmental toxins [11]. The repair mechanisms for nDNA are more efficient than mtDNA and over time, the accumulation of DNA damage can be associated with age-related vascular dysfunctions [12], which may correlate to the wet form of AMD. Increased DNA damage can also trigger apoptosis and autophagy [13], which can contribute to degenerative retinal diseases. The production of reactive oxygen/nitrogen species (ROS/RNS), such as superoxide radicals, hydrogen peroxide and peroxynitrites, are key factors as they lead to oxidative damage and apoptosis. Mitochondria are a major site of ROS production via the electron transport chain and because of this close proximity, the mtDNA is very susceptible to oxidative damage. Mitochondria are present in large numbers in retinal tissue since photoreceptors are one of the highest consumers of oxygen in the body. In 2006, Feher et al, reported significant decreases in numbers and size of mitochondria and an increase in peroxisomes in AMD retinas [14]. Harman theorized that mtDNA mutations and production of free radicals played a large role in the aging process [15].

The human mtDNA is a circular double stranded DNA that encodes 37 genes including 13 protein subunits essential for oxidative phosphorylation, and a non-coding region mtDNA D-loop which is critical for replication and transcription. The mtDNA have been classified into various haplogroups based on variable combinations of single nucleotide polymorphisms (SNPs) that have accumulated over 150,000 years. The oldest haplogroups have been identified from Africa and with eventual migration to other continents, mtDNA SNP variations due to adaptations to different environmental conditions evolved. At this time, geographic origins of populations can be identified by their haplogroup patterns. In general, the mtDNA SNP variations in coding and non-coding regions are associated with different rates of replication, transcription and metabolic processes [16] and it has been proposed that haplogroup-related variants may lead to differential responses for cell functions.

Both haplogroups H and J represent European-Caucasian populations. However, J haplogroup originated from northern Europe and the H haplogroup was from southern Europe. Recent studies have shown that individuals with the J haplogroup have higher susceptibility to developing AMD and that the H haplogroup is protective [17]. Our work, among others, has used the cybrid (cytoplasmic hybrid) model to demonstrate that the H and J haplogroups have differences in production of ATP, ROS and lactate levels, as well as cell growth rates and expressions of genes involved in the complement pathway [18]–[21]. However, it is not known if the different mtDNA haplogroups might influence the responses of the cells to stress factors.

In the present study, we have used the human RPE cybrids that contain either the H or J haplogroups to investigate their responses to environmental stress using UV-radiation, a potential risk factor for AMD. After exposure to sub-lethal dose of UV-radiation, the H cybrids and J cybrids, which have identical nuclei but differ only in their mtDNA haplogroups, show different rates of cell growth and expression levels for genes involved in inflammation, cell proliferation and apoptosis. Our findings support the hypothesis that in responses UV radiation, the inherited mtDNA variants (haplogroups) influence cells to have differential responses in major molecular pathways, which are associated with many aging diseases.

## Materials and Methods

### Ethics Statement

All research involving human participants was approved by the Institutional Review Board of University of California, Irvine (#2003-3131). Written informed consents were obtained and all clinical investigations were conducted according to the principles expressed in the Declaration of Helsinki.

### Transmitochondrial Cybrid Cultures

The preparations of transmitochondrial cybrids were performed as described in our previous manuscripts [22]–[24]. Cybrids were grown in DMEM/Ham’s F12 1:1 (Invitrogen-Gibco, Grand Island, NY) containing 24 mM sodium bicarbonate, 10% dialyzed fetal bovine serum and 1.0 mM sodium pyruvate. Mitochondrial haplogroups of each cybrid were confirmed by using PCR, restriction enzyme digestion and sequencing of mtDNA. All experiments utilized the H and J cybrids, which were at passage 5. Cybrids that contained mitochondria with haplogroup H (n = 3 different individuals) or J (n = 3 different individuals) were studied in 2 groups, the Control (untreated) group and Study group (treated with UV-radiation). Assays to determine the growth curve and gene expression analyses were performed for the individual cybrids in the Control and Study groups.

### UV-radiation Treatment

A sub-lethal dose of UV-radiation was defined as the dose sufficient to decrease the growth rate of cells but not cause irreversible damage to cell viability. Initially the cybrids were treated with 5, 10, and 20 mJ of UV-radiation using the Stratalinker 1800 (Stratagene, Santa Clara, CA) and analyzed for cell viability 48 and 96 hours later to identify the sub-threshold dose. 5 mJ UV-radiation did not have any impact on growth rate in the study group when compared to the untreated cells (data not shown). When cybrid cultures were treated with 20 mJ of UV exposure, there was significant loss of cells in both the H and J cybrids (data not shown). However, ninety-six hours after a single pulse of 10 mJ UV-radiation, we found that the growth rates of cells were temporarily reduced but they increased by 120 hours indicating that the UV radiation had a sub-lethal effect on the cells. Therefore, we chose 10 mJ UV-radiation as the dosage to treat the cybrids. Individual H or J cybrids were plated at 300,000 cells per well onto six well plates with 2 ml of culture media. Twenty-four hours after plating, the culture media were removed and the cybrids were treated with a single 10 seconds pulse of 10 mJ of UV-radiation using Stratalinker 1800. For the Control group, no treatment was given but culture media were changed at that time. Thereafter, culture media were changed every 48 hours in both groups.

### Growth Curve Assay

The growth curves of three different H cybrids and three different J cybrids in both Study and Control groups were analyzed over 6 days. For each individual cybrid, cells were plated and treated with UV-radiation as described above. Cells numbers were measured with the Cell Viability Analyzer (ViCell, Beckman Coulter, Miami, FL) using the trypan blue staining technique, which counts and averages cells over 50 images. The number of cells at the time of treatment (time-point 0 hours) was designated as 100% and percentage increase in growth of each cybrid were measured at 48, 96, 120 and 144 hours. The assays were run in triplicate and experiments were repeated twice. There were biological triplicates within each experiment.

### Isolation of RNA and cDNA Synthesis

RNA was isolated from both the Study and Control groups at 0 hours, 72 hours and 120 hours after UV exposure using the RNeasy Mini-Extraction kit (Qiagen, Inc.) as per the manufacturer’s protocol. The RNA was quantified using NanoDrop 1000 (Thermoscientific, Inc.). RNA was reverse transcribed into cDNA using the QuantiTect Reverse Transcription Kit (Qiagen, Inc.).

### Quantitative-PCR (Q-PCR) Analyses

In our previous publication, we demonstrated that CFH, C3 and EFEMP-1 genes were differentially expressed in H versus J cybrids (at 0 hours) [18]. In the present study, Q-PCR was performed using primers (QuantiTect primer assay, Qiagen) for genes associated with alternate complement pathway- Complement Factor-H (CFH), Decay Accelerating Factor for Complement (CD55/DAF), Complement Regulatory Protein (CD59), genes related to inflammation and cellular proliferation- Interleukin-33 (IL-33), Transforming Growth Factor, alpha (TGF-A), EGF containing fibulin like extracellular matrix protein 1 (EFEMP-1) and pro-apoptotic genes- Retinoic acid receptor, alpha (RARA), BCL2 binding component 3 (BBC3), BCL2-like 13 (BCL2L13) (Table 1). The Q-PCR was performed on individual H cybrids (n = 3) and J cybrids (n = 3) using a QuantiFast SYBR Green PCR kit (Qiagen) on a Bio-Rad iQ5 iCycler detection system. Gene expression levels were standardized for all primers using Hypoxanthine-guanine phosphoribosyltransferase (HPRT1, NM_000194) as a reference gene. Each sample at individual time-points was assessed in triplicate.

**Table 1:** Gene Functions and Accession Numbers.

Gene	Gene name	Gene Bank Accession No.	Function
CFH	Complement Factor H	NM_000186.	Essential in regulation of complement activation.
CD55/DAF	Decay accelerating factor forcomplement	NM_000574, NM_001114543,NM_001114544, NM_00111475.	Involved in regulation of complement cascade by accelerating decay of complement proteins and disrupting the cascade.
CD59	CD59 Molecule, ComplementRegulatory Protein	NM_000611, NM_203329,NM_203331, NM_001127223,NM_001127225, NM_001127226,NM_001127227.	Cell surface glycoprotein that regulates complement-mediated cell lysis, inhibits complement membrane attack complex and is involved in lymphocyte signal transduction.
IL-33	Interleukin 33	NM_033439, NM_001127180,NM_001199640.	Member of IL-1 family; Critical pro-inflammatory cytokine involved in production of T helper 2 associated cytokines.
TGF-A	TransformingGrowth Factor, alpha	NM_003236, NM_001099691.	Ligand for EGFR that activates signaling pathway for cell proliferation, differentiation and activation. It is also upregulated in human cancers.
EFEMP-1	EGF containing fibulin likeextracellular matrix protein 1	NM_004105, NM_018894,NM_001039348, NM_001039349.	High-risk gene associated with AMD, misfolded EFEMP-1 protein accumulates within RPE cells causing altered cellular function and inflammation. High EFEMP-1 levels are associated with tumor metastasis.
RARA	Retinoic acid receptor, alpha	NM_000964, NM_001145301,NM_001033603.	Nuclear retinoic acid receptor. Regulates transcription. Involved with apoptosis and differentiation.
BBC3	BCL2 binding component 3	NM_014417, NM_00112741,NM_001127240.	Member of BCL2 family, BH3 only pro-apoptotic subclass to induce mitochondrial outer membrane permeabilization, apoptosis, mitochondrial dysfunction and caspase activation.
BLC2L13	BCL2-like 13	NM_015367	Mitochondria specific protein whose over-expression results in apoptosis.

The selection of the genes analyzed in this study was based on our previous studies using the untreated H and J cybrids. Each of these genes were initially identified when cDNA from the H and J cybrids were analyzed by the Affymetrix Human U133 Plus 2.0 Array, which is a comprehensive human genome expression array that allows for the analyses of over 40K transcripts. Then differences between the individual H and individual J cybrids were verified by Q-PCR analyses [22], [24]. The CD59 were analyzed because it is a major inhibitor of the alternative pathway.

### Statistical Analysis

Data were subjected to statistical analysis by ANOVA using GraphPad Prism 5.0 version statistics program (GraphPad Software Inc., San Diego, CA). For the growth curve assays, the Control samples were normalized to 100% and then the cell count for the 48, 96, 120 and 144 hours samples were calculated accordingly. In the gene expression assays, the ΔCt was calculated as the difference between the Ct (threshold cycle) of primer and Ct of the housekeeper/reference gene. The ΔΔCt was calculated as the mean difference of ΔCts of a specific primer at a single time point between H and J cybrids or ΔCts of the primer being compared at two different time points of either H or J cybrids. The fold values were calculated using the formula Fold = 2^−ΔΔCt^. Fold values were calculated relative to the readings for corresponding H cybrids at 0 hours. Data is presented as mean ± standard error of mean (SEM). Experiments were performed in triplicate. *P* values <0.05 (two-tail test) were considered statistically significant.

## Results

### Growth Curve Assay

**Figure 1:**
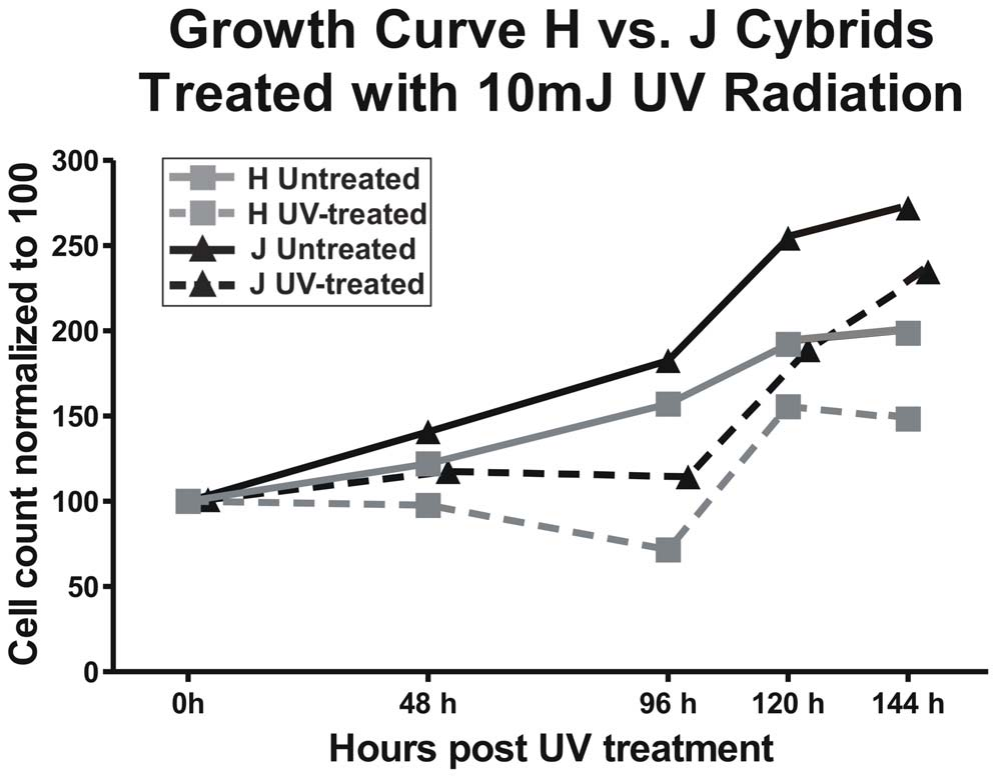
Differential growth patterns were observed for H cybrids versus J cybrids in UV-treated and untreated groups. Cybrids containing J haplogroup mtDNA showed a steeper slope for the growth curve than H haplogroup mtDNA in both UV-treated and untreated groups. In untreated there were constant increases in growth patterns for both H and J cybrids. In the UV-treated samples, the growth rates slowed or declined until approximately 96 hours post treatment, and then increased again. Triplicate wells were run for each sample and the experiments were repeated twice. During the growth curve assay, each sample was run in triplicate. Grey solid line, untreated H cybrids; Grey broken line, UV-treated H cybrids; Black solid line, untreated J cybrids; Black broken line, UV-treated J cybrids.

The 0 hour cell counts were normalized to 100% in the both Control (untreated) and Study (UV-treated) groups (Figure 1). At each time point, J-untreated cybrids showed greater growth than H-untreated cybrids (48 hours- 141% vs. 122%, 96 hours- 183% vs. 157%, 120 hours- 254% vs. 192%, and 144 hours- 272% vs. 198%). In the UV-treated groups at time interval between 48 and 96 hours, there was initially a downslope followed by an upward trend in the growth patterns. The J-treated cybrids showed higher growth rates than H-treated cybrids (48 hours-116% vs. 98%, 96 hours- 113% vs. 72%, 120 hours- 188% vs. 156%, and 144 hours- 234% vs. 148%), but the growth rates were reduced in comparison to their corresponding untreated groups.

### Expression of Genes Normalized to 0-Hour H Cybrids

#### Complement pathway inhibition genes

**Figure 2:**
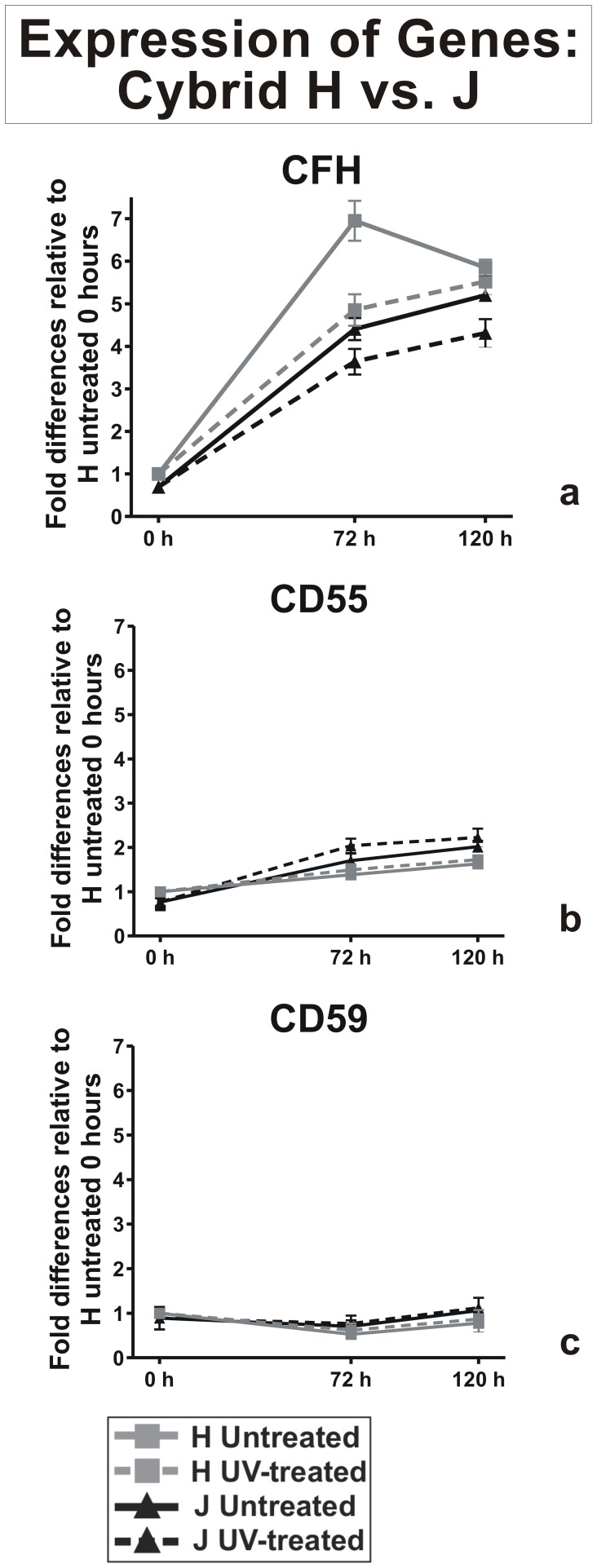
Expression patterns of alternate complement inhibitor genes after UV treatment. The 0-hour value for H-untreated cybrid was normalized to 1 and then compared to gene expression values at 0, 72 and 120 hours. Panel 2a: At 0-hour, the J-untreated cybrids had lower levels of CFH compared to H-untreated cybrids (p = 0.003). At 72 hours and 120 hours, the H-untreated cybrids (grey solid line) showed higher levels of CFH expression compared to the J-untreated cybrids (black solid line). After UV exposure, the H-treated cybrids (grey broken line) expressed increased CFH levels compared to the J-treated cybrids (black broken line) at both 72 and 120 hours. Panel 2b: At 0-hours the H-untreated cybrids showed higher expression levels for CH55 (grey solid line) compared to J-untreated cybrids (black solid line, p = 0.002). At 72 and 120 hours post-UV treatment, there were no significant differences in the CD55 expression levels. Panel 2c: The CD59 expression levels were similar for the untreated (grey solid and black solid lines) and UV-treated (grey broken and black broken lines) H and J cybrids. There were triplicate wells for each sample and the experiments were repeated twice. n = 3 different individuals for the H cybrids and n = 3 different individuals for the J cybrids.

Initially we normalized the H-untreated cybrids at 0 hour value to 1 and then compared the H and J cybrid values of gene expression at 0, 72 and 120 hours (Figure 2). At 0 hours, we found that the H-untreated cybrids had significantly higher expression levels of CFH than J-untreated cybrids (1 vs. 0.69±0.05 fold, p = 0.003). In the H-untreated cybrids, there was an increase in CFH gene expression at 72 hours (6.9±0.19 fold, p<0.0001) and 120 hours (5.85±0.07 fold, p<0.0001) compared to the 0 hours H-untreated cybrids. The J-untreated cybrids started at a lower level of CFH expression (0.69±0.05 fold, p<0.01) and increased to 4.4±0.1 fold, p<0.0001 and 5.2±0.03 fold, p<0.0001 at 72 and 120 hours, respectively. Compared to the 0-hour H cybrids, the H-treated cybrids cultured 72 hours and 120 hours had increased levels of CFH expression (4.8±0.15 fold, p<0.001 and 5.52±0.12 fold, p<0.0001, respectively). At 72 hours and 120 hours, the J-treated cybrids also showed an increase expression of CFH compared to the 0-hour H-untreated cybrids (3.63±0.2 fold, p<0.001 and 4.31±0.13 fold, p<0.0001), respectively (Figure 2a).

At 0-hours, the CD55 gene expression levels were significantly higher in the H-untreated cybrids versus J-untreated cybrids (1.00 vs. 0.77±0.05 fold, p = 0.002), however, both H and J cybrids did not increase greatly after UV treatment (Figure 2b).

The CD59 expression levels at 0-hours were not significantly different between H-untreated and J-untreated cybrids (1 vs. 0.89±0.10 fold, p = 0.30) or after 120 hours of UV treatment (0.86±0.08 fold, p = 0.14 vs 1.12±0.04 fold, p = 0.06) (Figure 2c).

#### Inflammation, cellular proliferation and angiogenesis genes

**Figure 3:**
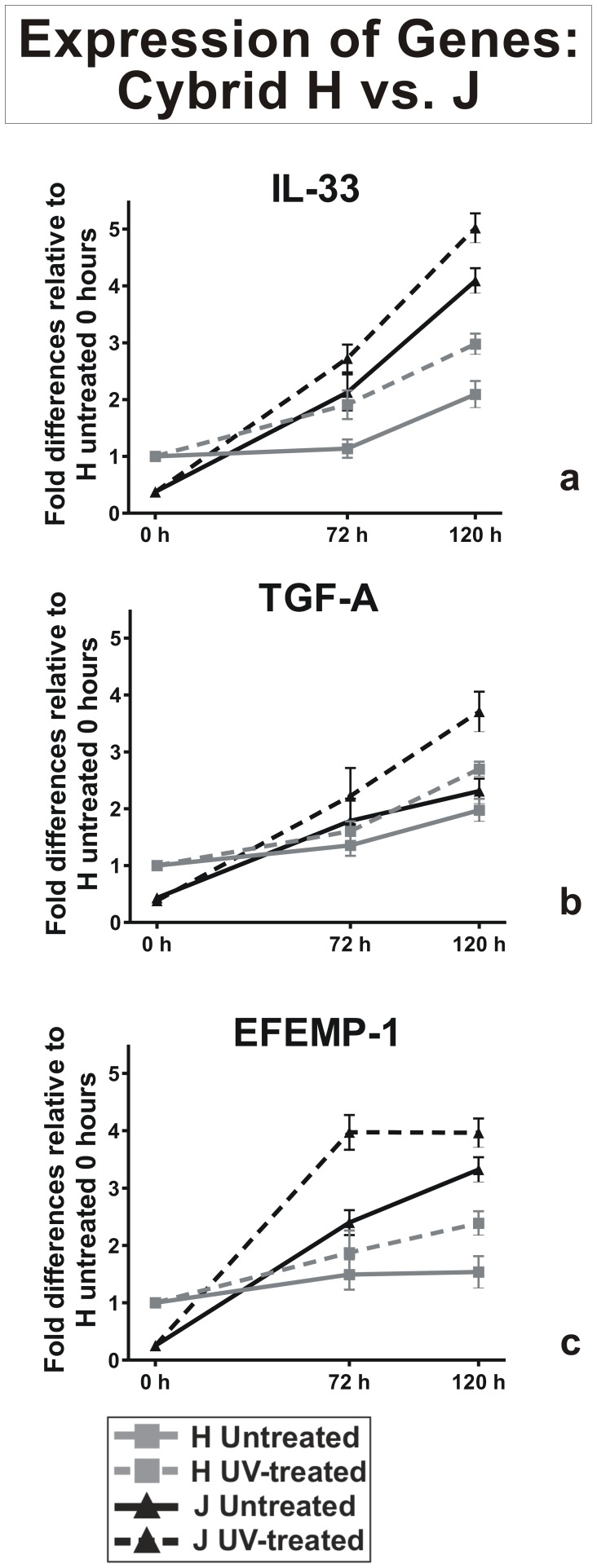
Expression patterns of inflammation, cellular proliferation and angiogenesis genes after UV treatment. The 0-hour values for H-untreated cybrids were normalized to 1 and then compared to gene expression values at 0, 72 and 120 hours. Panel 3a: At 0-hour, the J-untreated cybrids (black solid line) had lower expression levels of IL-33 compared to H-untreated cybrids (grey solid line, p<0.0001). At 120 hours, the H-untreated cybrids (grey solid line) showed higher levels of IL-33 expression compared to the J-untreated cybrids (black solid line). At both 72 and 120 hours, the J-treated cybrids showed higher levels of IL-33 expression compared to the H-treated cybrids. Panel 3b: At 0-hours, the TGF-A expression levels were lower for the J-untreated cybrids (black solid line) compared to the H-untreated cybrids (grey solid line, p<0.0001). At 120 hours after UV-treatment, the TGF-A levels for both H-treated (grey broken line) and J-treated (black broken line) were higher than the untreated H (grey solid line) or J (black solid line) cybrids. Panel 3c: At 0-hours, the expression levels for EFEMP1 were lower in the J-untreated (black solid lines) compared to H-untreated cybrids (grey solid lines, p = 0.0001). By 120 hours, the J-treated cybrids (black broken line) and J-untreated cybrids (black solid line) showed greater fold increases of EFEMP1 expression compared to the H-treated (grey broken line) and H-untreated (grey solid line) cybrids. There were triplicate wells for each sample and the experiments were repeated twice. J cybrids, n = 3 different individuals; H cybrids, n = 3 different individuals.

With this category of genes, we again normalized the H cybrids at 0-hours to a value of 1 and then compared this against all other values (Figure 3). At 0-hours, Q-PCR analyses showed lower expression levels in J-untreated cybrids for IL-33 (0.37±0.01 fold, p<0.0001) compared to the 0-hour H cybrids. At 120 hours, the J-untreated cybrids and the H-untreated cybrids showed significantly elevated levels of IL-33 compared to the 0-hour H cybrids (4.09±0.09 fold, p<0.001 and 2.09±0.09 fold, p<0.001, respectively, Figure 3a). At 120 hours after UV treatment, the IL-33 expression in the J-treated cybrids increased to 5.01±0.1 fold (p<0.001) while the H-treated cybrids increased 2.97±0.07 fold (p<0.001).

A similar pattern was seen for the TGF-A expression levels, with the J-untreated cybrids having lower levels than the H-untreated cybrids at 0 hours (0.38±0.02 vs. 1 fold, p<0.0001, Figure 3b). At 120 hours, the J-untreated (2.26±0.08 fold, p<0.001) and H-untreated cybrids (1.97±0.08 fold, p = 0.04) showed an elevated expression levels of TGF-A compared to the 0-hour H-untreated cybrids. After UV treatment, there was an increase for TGF-A expression in the J-treated cybrids (3.71±0.14 fold, p<0.0001) and H cybrids, (2.7±0.05 fold, p<0.0001) compared to the 0-hour H cybrids.

At 0-hour, Q-PCR analyses showed lower expression levels for EFEMP-1 in J-untreated cybrids (0.26±0.02 fold, p = 0.001) compared to the 0-hour H cybrids (Figure 3c). At 120 hours, compared to the 0-hours H cybrids, the EFEMP-1 gene expression for both the J-untreated cybrids, (3.3±0.17 fold, p<0.0001) and J-treated cybrids (4.0±0.09 fold, p<0.0001) increased to a greater degree than H-untreated cybrids (1.54±0.11 fold, p<0.0001) and H-treated cybrids (2.39±0.08 fold, p<0.0001).

#### Expression levels for apoptosis genes

**Figure 4:**
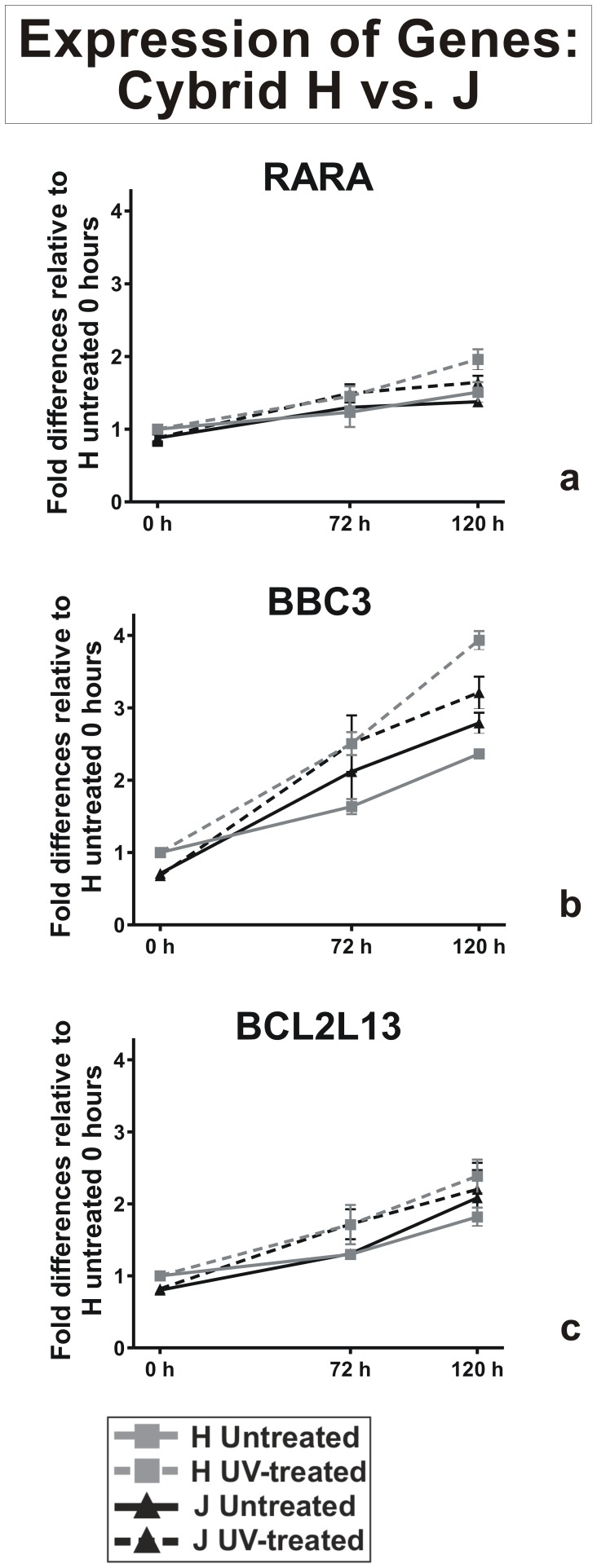
Expression patterns of pro-apoptosis genes after UV treatment. The 0-hour values for H-untreated cybrids were normalized to 1 and then compared to gene expression values at 0, 72 and 120 hours. Panel 4a: At 0-hour, the J-untreated cybrids (black solid line) had lower expression levels of RARA compared to H-untreated cybrids (grey solid line, p = 0.0036). Panel 4b: At 0-hour, the J-untreated cybrids (black solid line) had lower expression levels of BBC3 compared to H-untreated cybrids (grey solid line, p<0.01). At 120 hours after UV treatment, the H-treated cybrids (grey broken line) and J-treated cybrids (black broken line) showed higher expression levels of BBC3 compared to H-untreated (grey solid line) and J-untreated (black solid line). Panel 4c: At 0-hours, the J-untreated cybrids (black solid line) showed lower expression levels of BCL2L13 compared to the H-untreated cybrids (grey solid line, p = 0.002). After 120 hour of UV treatment, the H-treated (grey broken line) and J-treated (black broken line) cybrids showed an increase in BCL2L13 expression levels. There were triplicate wells for each sample and the experiments were repeated twice.

We analyzed 3 pro-apoptotic genes and found that only RARA and BBC3 showed significant increases after UV treatment (Figure 4). All samples were normalized to the H-untreated cybrid at time point 0-hours. The gene expression levels for the 0-hour J-untreated cybrids were significantly lower compared to the 0-hour H-untreated cybrids (RARA, 0.88+0.03 fold, p = 0.003, Figure 4a; BBC3, 0.71±0.2 fold, p<0.01, Figure 4b; and BCL2L13; 0.81±0.05 fold, p = 0.002, Figure 4c). At 120 hours after UV treatment, the H-treated and J-treated cybrids showed increased expression levels for RARA (1.96±0.05 fold, p<0.001 and 1.64±0.03 fold, p = 0.009, respectively); BBC3 (3.78±0.15, p<0.001 fold and 3.21±0.09 fold, p<0.0001); and BCL2L13 (2.39±0.05 fold, p<0.001 and 2.20±0.15 fold, p<0.0001, respectively) compared to the 0-hour H-untreated cybrids.

### Comparison of Gene Expression Levels from 0 to 120 Hours

**Figure 5:**
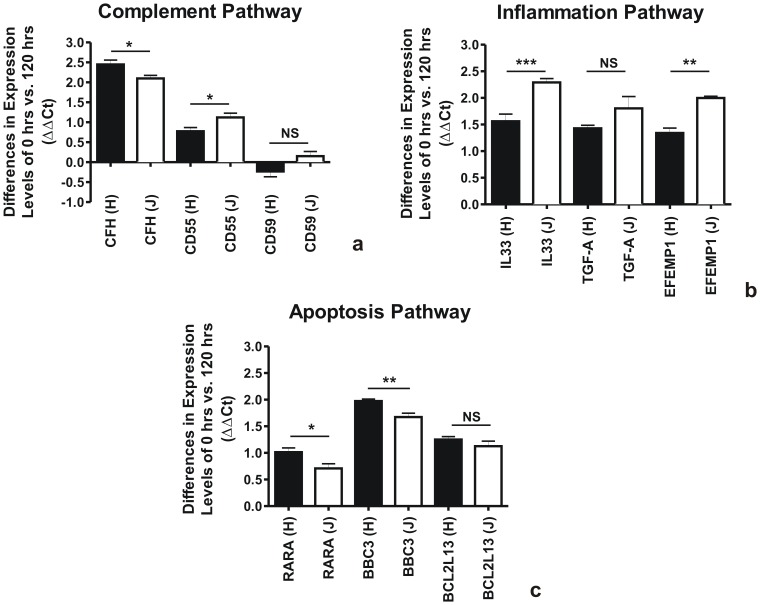
Comparison of gene expression levels from 0 to 120 hours after UV treatment. The solid black bars represent the ΔΔCt values of H-untreated cybrids at 0 hour compared to H-treated cybrids at 120 hours. The open bars represent the ΔΔCt values of the J-untreated cybrids at 0 hours compared to J-treated cybrids at 120 hours. Panel 5a: At 120 hours after UV treatment, the H-treated cybrids have higher expression levels for CFH compared to the J-treated cybrids (p<0.02) but lower expression for the CD55 gene (p<0.03). There was no significant difference for the CD59 levels in the H-treated versus the J-treated cybrids. Panel 5b: At 120 hours after UV treatment, the J-treated cybrids have higher expression levels for IL-33 and EFEMP1 genes compared to the H-treated cybrids (p<0.02) but similar expression levels for the TGF-A gene. (p = 0.14). Panel 5c: At 120 hours after UV treatment, the H-treated cybrids have higher expression levels for RARA (p = 0.02) and BBC3 (p = 0.004) genes compared to the J-treated cybrids. There expression levels for the BCL2L13 gene were similar for both cybrids (p = 0.28).

The analyses performed above compared the gene expression values for all samples to the 0-hour H-untreated cybrids. However, in all cases the 0-hours J-untreated cybrids showed lower gene expression levels at baseline than the 0-hour H-untreated cybrids. Therefore, in order to measure the total expression increase of each of the genes, we needed to compare the values at the 120-hour period compared to the original 0-hour baseline for the H cybrids and J cybrids (represented as ΔΔCt values). Figure 5 shows the ΔΔCt values of the 120-hour UV-treated H cybrids compared to the 0-hour H-untreated cybrids and the UV-treated 120-hour J cybrids compared to the 0-hour J-untreated cybrids.

At 120 hours after UV treatment, there was a significant increase in CFH gene expression from their respective baseline levels in the H-treated cybrids compared to the J-treated cybrids (ΔΔCt 2.44±0.11 vs. ΔΔCt 2.09±0.07, p<0.02, Figure 5a). The ΔΔCt values for CD55 expression were decreased in the H-treated cybrids compared to the J-treated cybrids, (ΔΔCt 0.78±0.09 vs. ΔΔCt 1.12±0.11, p<0.03, Figure 5a). There were no significant differences in ΔΔCt values of CD59 expression for the H-treated cybrids compared to J-treated cybrids (ΔΔCt −0.23±0.2 vs. ΔΔCt 0.15±0.13, p = 0.064).

The mean ΔΔCt values in the H-treated cybrids were significantly lower for the IL-33 gene (1.56±0.12, p<0.001 vs. 2.29±0.1, p<0.0008) and the EFEMP-1 gene (ΔΔCt 1.34±0.004 vs. ΔΔCt 1.99±0.03, p<0.001) compared to the J-treated (Figure 5b). There were no statistically significant differences in ΔΔCt values for the TGF-A expression (H cybrids value was ΔΔCt 1.43±0.05 vs. J cybrids value of ΔΔCt 1.8±0.22, p = 0.14,). This demonstrates that when H and J cybrids were treated with the same amount of UV radiation under identical conditions, the J-treated cybrids showed significantly greater IL-33 and EFEMP1 expression levels from baseline, when compared to H-treated cybrids. However, the increases in TGF-A gene expression levels from their respective baseline values were quantitatively similar in both H-treated and J-treated cybrids.

In Figure 5c, we analyzed the expression levels for the three pro-apoptosis genes comparing H-treated cybrids to J-treated cybrids at 120 hours. The J-treated cybrids consistently showed lower mean change in ΔΔCt values compared to the H-treated cybrids for the RARA (ΔΔCt 0.71±0.07 vs. ΔΔCt 1.01±0.03, p = 0.02) and BBC3 genes (ΔΔCt 1.67±0.06 vs. ΔΔCt 1.94±0.04, p = 0.004) but not the BCL2L13 gene (ΔΔCt 1.12±0.07 vs. ΔΔCt 1.25±0.05, p = 0.28). This indicates that after UV radiation, the H-treated cybrids showed greater increases of expression levels of pro-apoptotic genes than J-treated cybrids.

**Figure 6:**
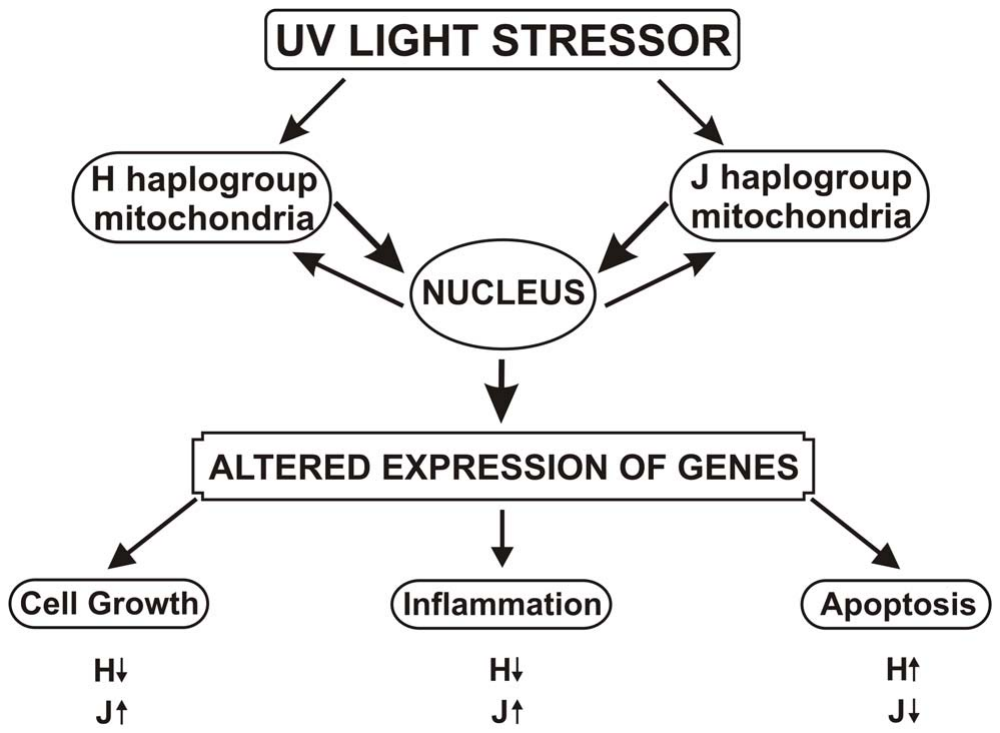
Schematic summary of the changes found in cybrids after UV treatment. In response to identical UV conditions, cybrids with identical nuclei but different mtDNA haplogroups (H vs. J) showed differential responses of cell growth and expression patterns genes related to inflammation and apoptosis. After UV treatment, the J-treated cybrids had increased cell growth rates but a gene expression pattern consistent with higher levels of inflammation (elevated IL-33 and EFEMP1 along with decreased expression of CFH, a complement pathway inhibitor) compared to H-treated cybrids. The pro-apoptosis genes (RARA and BBC3) were expressed at a lower level in J-treated cybrids after UV treatment.

In summary, our findings demonstrate that at different time periods after UV sub-lethal radiation, the J cybrids maintains higher rates of growth, higher expression levels for inflammation genes but lower expression levels for apoptosis-related genes (Figure 6).

## Discussion

The study investigates the response of cybrids, with identical nuclei but either H haplogroup mtDNA or J haplogroup mtDNA, to sub-lethal UV-radiation, which is an oxidative stressor and a risk factor for AMD [25]. Our previous study has shown that J cybrids have higher growth rates than H cybrids when cultured in similar environmental conditions [18]. In addition, we reported that although both H and J cybrids have similar copy numbers of mtDNA, H cybrids produce higher ATP levels and utilize oxidative phosphorylation (OXPHOS) while the J cybrids utilize predominantly glycolysis [18].

In our study, we observed that J haplogroup cybrids, with or without UV treatment, had more rapid growth rates even though the J cybrids are less energy efficient [18]. After 144 hours in vitro, the J-untreated cybrids had approximately 75% higher numbers of cells than the H-untreated cybrids. After UV treatment, the J-treated cybrids showed an approximately 85% higher cell count levels compared to the H-treated cybrids. The increased growth rates may be related in part to increased expression of IL-33 and EFEMP-1 genes, which play key roles in cell proliferation. The mechanisms by which the J haplogroup mtDNA affects rapid growth rates and glycolysis are not understood. One can speculate that the phenomenon might be similar to Warburg effect, described in 1956 by Dr. Otto Warburg [26], which suggested that tumor cells with damaged respiratory mechanisms have uncontrolled proliferation. These cancer cells had adapted to hypoxic conditions by shifting to glycolysis for ATP production, and even if returned to normoxic conditions, the cells continued with glycolysis as their main source of energy production. Similarly, the J haplogroups represent a subgroup of the Caucasian population which originated in Northern Europe, and over thousands of years, in response to extremely cold climatic conditions, may have developed adaptations within the mtDNA which preferentially uses glycolysis rather than OXPHOS. Others have shown that J haplogroups have accumulated higher levels of non-synonymous SNPs compared to other haplogroups, suggesting either a high mutation rate or a continuous selection process in the lineage [27]. We speculate these divergent energy efficiencies, as seen in the J versus H haplogroups, may influence the mitochondrial-nuclear interactions, thereby causing different expression levels of genes in major pathways. Further studies are underway to clarify the relationships between mtDNA variants and nuclear responses.

Complement system activation and inflammation are major events contributing to AMD pathogenesis [28]. Studies have reported accumulation of immunoglobulin and complement components within drusen [29]–[31], associations between genetic variants of CFH, C2/CFB, C3, and CFI with increased risk for AMD [32], [33], and elevated serum CRP levels in AMD patients [34]. Studies of European mtDNA variants have revealed that J, T and U haplogroups are associated with AMD [17], [35]–[38] while the H haplogroup is protective [39]. Our previous paper shows that at resting state, the J cybrids express lower levels of CFH than H cybrids. After UV-treatment, J cybrids still showed lower CFH levels at 72 and 120 hours in both the untreated and UV-treated samples. Since CFH is a negative regulator of the alternative complement pathway, the lower levels in J haplogroup subjects would likely lead to increased activation of the complement pathway and higher levels of inflammation. Alternatively, the higher CFH levels in H cybrids, as seen at all time points, may be associated with the protective effects associated between H haplogroup and AMD. In cells from the outer retinal layers, photo-oxidative stress induces complement activation and decreases the level of CFH, which results in increased inflammatory responses [40]. Interestingly, we found less than 2-fold increase of CD55 expression after UV treatment, suggesting that CFH was the major inhibitor mediated by mtDNA. This makes the CFH gene an attractive target for future therapies to treat AMD and other complement-related diseases.

RPE cells, with their high oxygen demands and metabolic rates [41], are susceptible to UV radiation, which can cause loss of cell viability, mitochondrial dysfunction, DNA damage and apoptosis [42]. UV exposure can increase ROS production in RPE cells that can trigger inflammation [43]–[45]. In our study, we found similar expression patterns for IL-33 and EFEMP-1 genes, whereby at resting stages, the J-untreated cybrids had levels lower than H-untreated cybrids but after UV stress, the J-treated cybrids showed increased expression levels of each gene compared to H-treated cybrids. This is important because these genes play a role in inflammation, innate immunity and/or angiogenesis, which are pathologic processes of AMD. IL-33, which can be found in RPE cells, is a cytokine belonging to the IL-1 superfamily, and can regulate inflammatory cytokines including IL-6, IL-8, IL-1B and TNF-alpha. Alipoprotein E (ApoE), a high-risk gene associated with AMD, mediates the distribution of lipids and cholesterol. Abnormal functions of ApoE can lead to hypercholesteremia [46], and increased production of amyloid, a protein associated with both AMD (geographic atrophy) and Alzheimer’s disease. It has been reported that in RPE cells, beta-amyloid can stimulate IL-33 mediated inflammatory responses [47], which suggests that there may be a correlation between amyloid production, IL-33 and ApoE levels related to RPE cells responses to oxidative stressors.

TGF-A plays a role in both angiogenesis and wound healing [48]. In addition, elevated levels of TGF-A have been consistently observed in gastrointestinal cancers, breast cancers and primary epithelial ovarian cancers [49]–[51]. While an association between TGF-A and AMD has not been reported, it is a relatively newly described gene and little is known about its functions. It is worth noting that the TGF-A and IL-33 may interact at some level because their expression patterns closely followed each other in our experiments.

Increased expression of EFEMP-1 genes was also found in untreated and UV-treated J cybrids. EFEMP-1 is another important cancer-related gene that was differentially expressed in UV-treated J cybrids. EFEMP-1, a favorable prognostic marker for glioblastoma multiforme tumors [52], modulates the extracellular environment and influences important signaling pathways. Song and coworkers transfected EFEMP-1 into HeLa cells and observed increased VEGF expression, acceleration of angiogenesis, increased growth and proliferation of cervical carcinoma *in vivo* [53]. It is reported that misfolded EFEMP-1 accumulates within endoplasmic reticulum and up-regulates VEGF expression in pancreatic adenocarcinoma [54]. Therefore, the finding that EFEMP-1 expression is mediated by mtDNA haplogroups is exciting because it implies that the mtDNA can greatly influence this important pathway and that it is responsive to sub-lethal UV radiation.

With respect to AMD, EFEMP-1 expressed in RPE cells is a high-risk gene associated with AMD and Doyne honeycomb retinal dystrophy. Misfolded EFEMP-1 protein accumulates within RPE cells causing altered cellular function and inflammation [55]. EFEMP-1 protein is present between RPE layer and drusen [56], [57]. Roybal et al. studied the response of transfected EFEMP-1 wild type and mutant EFEMP-1 R345W on VEGF expression in ARPE-19 cell line. R345W mutation in EFEMP-1 is linked to two inherited early–onset macular degenerative retinal diseases Malattia Leventinese (ML) and Doyne honeycomb retinal dystrophy (DHRD). There was an abnormal accumulation and retention of EFEMP-1 R345W in the endoplasmic reticulum that induced the unfolded protein response (UPR) activation followed by increased VEGF expression. Activation of UPR and VEGF expression may be correlated with RPE dysfunction and choroidal neovascularization in AMD patients [58]. In our study, the UV-treated J cybrids showed a 4-fold increase in EFEMP-1 levels while the UV-treated H cybrids had a 2.56-fold increase. Even though the mechanisms by which EFEMP-1 might contribute to AMD is not known, based upon the cancer studies, it is reasonable to speculate that numerous downstream pathways are activated by increased EFEMP-1 levels. Most importantly, it must be pointed out that (1) the J and H cybrids showed a differential response to the UV treatment, and (2) these cybrids differed only in their mtDNA but had identical nuclei and treatment conditions. This is important because it strongly suggests that within an individual, the mtDNA haplogroup background may provide a baseline for expression of major signaling pathways, which are then influenced to different degrees by identical stressors, depending upon which haplogroup the individual possesses. One can speculate that this may explain in part why some individuals are more susceptible to environmental stressors, such as smoking or UV light, while others are not.

In our study, we investigated expression levels for three apoptosis-related genes and found significant changes in RARA and BBC3. In general, BBC3 is involved in BAX-dependent cell death related to developing retina and brain [59] but in the adult nervous system the cell death process is mediated by BIM. BBC3 expression can be altered in response to various insults and is involved in adult retinal cell degeneration [60]–[62]. In a mouse model, UV exposure can increase BIM expression, which in turn can increase BBC3 expression and caspase-dependent cell death in retinal photoreceptor cells [63]. Our study demonstrates a 3.78-fold increase expression for BBC3 in UV-treated H cybrids occurring in response to UV-radiation stress. We need to be cautious because although the BBC3 expression levels are elevated, this may or may not translate into higher apoptotic activities in the UV-treated H cybrids.

A second apoptotic gene, RARA, was differentially expressed in the H versus J cybrids after UV treatment. RARA, which is present in the bipolar cells and retinal ganglion cells, is an important regulator for development [64]. In addition, RARA upregulates expression of glial cell-derived neurotrophic factors (GNDF), which inhibits vascular permeability factor-VEGF [65]. We observed increased expression of RARA in UV-treated H cybrids as compared to J cybrids. If RARA indirectly inhibits VEGF expression, then this may correlate with lower incidence of wet AMD in individuals having H haplogroup mtDNA.

The cybrid model using different Rho^0^ cells as the host cell line has provided similar findings to ours by showing that the mtDNA haplogroup profiles influence cellular/molecular responses. After H_2_O_2_–induced oxidative stress, the SIRT3 gene was downregulated in osteosarcoma cybrids with the J haplogroup compared to H cybrids [66]. Lin and coworkers used cybrids created with Taiwanese haplogroups and showed that the B4b haplogroup had increased susceptibility to H_2_O_2_ compared to other ethnic Chinese backgrounds. Bellizzi and co-workers used the osteosarcoma cybrid model to demonstrate that at baseline the H cybrids had significantly lower levels of heat shock protein (HSP)60 and HSP75 but after oxidative stress, the H cybrids accumulated HSP60 within mitochondria at a much greater rate than other cybrids [67]. In another series of experiments, they showed that in basal and H_2_O_2_-stressed conditions, the H and J cybrids showed different levels of interleukin (IL)-6, IL-1beta and tumor necrosis factor receptor 2, which are stress–related cytokines [68]. Finally, an osteosarcoma cybrid model showed that the H cybrids differed significantly from Uk cybrids with respect to mitochondrial RNA levels, protein synthesis, membrane potential and growth capacity [69]. These findings, along with ours, strongly support our hypothesis that mtDNA haplogroups can mediate molecular pathways that influence the complement/inflammation responses of cells.

## Conclusions

Our findings support the hypothesis that nuclear-mitochondrial interactions play significant roles in modulating complex phenotypic responses to sub-lethal UV radiation. We found that mtDNA variability, as represented by H versus J haplogroups, can influence genetic expressions of cells after UV stress. Increasing our understanding of nuclear-mitochondrial interactions, and their relationship to environmental stimuli, will lead to an entirely new era of disease management in which the mitochondria, with its unique DNA, will become a target for therapy.
